# Hydrophobic Modification of Nanocellulose via a Two-Step Silanation Method

**DOI:** 10.3390/polym10091035

**Published:** 2018-09-18

**Authors:** Wensheng Lin, Xiaoyong Hu, Xueqing You, Yingying Sun, Yueqin Wen, Wenbin Yang, Xinxiang Zhang, Yan Li, Hanxian Chen

**Affiliations:** 1 College of Materials Engineering, Fujian Agriculture and Forestry University, Fuzhou 350002, China; wensheng0817@163.com (W.L.); hxy1198578262@163.com (X.H.); 15880884064@163.com (X.Y.); 18065167449@163.com (Y.S.); fafuwyq@163.com (Y.W.); fafuywb@163.com (W.Y.); xxzhang0106@163.com (X.Z.); 2 College of Light Industry, Textile and Food Engineering, Sichuan University, Chengdu 610065, China

**Keywords:** nanocellulose, hydrophobicity, dodecyltrimethoxysilane, silanation, KH560

## Abstract

Dodecyltrimethoxysilane (DTMOS), which is a silanation modifier, was grafted onto nanocellulose crystals (NCC) through a two-step method using KH560 (*ɤ*-(2,3-epoxyproxy)propytrimethoxysilane) as a linker to improve the hydrophobicity of NCC. The reaction mechanism of NCC with KH560 and DTMOS and its surface chemical characteristics were investigated using Fourier transform infrared spectroscopy (FTIR), X-ray photoelectron spectroscopy (XPS) and HCl–acetone titration. These analyses confirmed that KH560 was grafted onto the surface of NCC through the ring-opening reaction, before DTMOS was covalently grafted onto the surface of NCC using KH560 as a linker. The grafting of NCC with DTMOS resulted in an improvement in its hydrophobicity due to an increase in its water contact angle from 0° to about 140°. In addition, the modified NCC also possessed enhanced thermal stability.

## 1. Introduction

As one of the most abundant organic homopolymers on Earth, nanocellulose crystals (NCC) have advantages of widespread availability [1], exceptional mechanical properties [2], high aspect ratio [3] and low density [4]. This makes it a potential candidate for reinforcements in bionanocomposites [5]. In addition, due to its very high crystallinity, NCCs have a high elastic modulus of 120–170 GPa, which is greater than the other organic materials [6]. Nevertheless, before it can be a good reinforcement for bionanocomposites, the poor interface compatibility between NCC and polymeric matrix should be settled [1]. This problem mainly results from the difference in the hydrophobicity of the NCC and polymeric matrix. NCC is quite hydrophilic, while most of polymeric matrices are hydrophobic [7]. The chemical modification of NCC is an effective method for improving the interface compatibility of the subsequent bionanocomposites. The modification method could be classified into the following types: isocyanation [7], esterification [8], silanation [9,10,11], oxidation [12,13], acylation and so on [14]. For example, Siqueira et al. [7] prepared hydrophobic NCCs by n-octadel isocyanate (C_18_H_37_NCO) modification. The modified sample displayed a homogeneous dispersion in organic solvents. Furthermore, de Menezes et al. [15] reported an esterification method of NCC, which used fatty acid chlorides. The modifiers with different aliphatic chains (C_6_H_13_–, C_12_H_25_–, and C_18_H_37_–) had different hydrophobic modification effects on NCCs.

Among these methods, the silanation method has been widely applied for the hydrophobic modification of NCC [10,16,17]. These silane modifiers include both hydrophobic groups and functional groups, such as vinyl, amino, isocyanate, chloride and alkoxy. The most widely used silane modifier is the alkoxysilanes. In the presence of water and catalysts, the hydrolysis of alkoxy groups takes place, which generates silanol groups. The self-condensation of silanol groups leads to the formation of flexible polysiloxane chains. These hydrophobic polysiloxane chains will be physically adsorbed onto the surface of NCC through hydrogen bonds and it is difficult to remove them by extraction [18]. In some studies, the formation of Si–O–C bonds was reported due to the dehydration of –C–OH of cellulose and silanol groups of the silane modifier [17]. However, unlike C–O–C and Si–O–Si bonds, the Si–O–C bond is not stable for hydrolysis with a catalyst. NCCs and alkoxysilanes seem to be combined by physical adsorption rather than covalent bonding. As a result, the lack of covalent bonds would limit the effects of the coupling on the mechanical strength of the bionanocomposites [19]. The intermolecular forces will be easily destroyed during the melt extrusion process, which leads to dissatisfactory mechanical strength of composites [20]. In addition, even if the –Si–O–C– bonds between silane modifier and NCC were formed, the self-condensation of the silane modifier cannot be avoided. There are also a considerable number of polysiloxane chains adsorbed onto the surface of NCC. Therefore, stable covalent bonding between NCC and alkoxysilanes is of benefit for its application in the reinforcement of bionanocomposites.

In this paper, a two-step silanation method was proposed for surface hydrophobic modification of NCC by using DTMOS as a hydrophobic modifier and KH560 as a linker between NCC and DTMOS. KH560 was grafted onto NCC by –C–O–C– bonds, which were created by a ring-opening reaction of epoxy groups of KH560, before the DTMOS was grafted onto the KH560 modified NCC by –Si–O–Si bonds. Finally, the modified NCC with improved hydrophobicity and thermal stability was obtained, before the properties of NCC before and after modification were systematically investigated. The very hydrophobic –C_12_H_25_ groups were grafted onto the surface of NCC by –C–O–C– and Si–O–Si– bonds in this two-step silanation method. This method is totally different from the reported methods in which instable –Si–O–C– bonds were formed.

## 2. Experimental Section

### 2.1. Materials

Microcrystalline cellulose (MCC) was purchased from Huzhou Yinhuxinwang, Chemical Co., Ltd., Fuzhou, China. Sulfuric acid (H_2_SO_4_), HCl (0.15 mol L^−1^) and acetone were supplied by Sinpharm Chemical Reagent Co., Ltd., Shanghai, China. Dioxane, triethylamine, phenolphthalein and ethanol were obtained from Tianjin Zhiyuan Chemical Reagent, Co., Ltd., Tianjin, China. NaOH, while *ɤ*-(2,3-epoxyproxy)propytrimethoxysilane (KH560) was obtained from Xilong Chemical Co., Ltd., Shanghai, China and Jinan Xingfeilong Chemical Co., Ltd., Shandong, China, respectively. Dodecyltrimethoxysilane (DTMOS) was purchased from Tokyo Chemical Industry, Tokyo, Japan. All chemicals were used as received without further purification.

### 2.2. Production of NCC

NCC was prepared by sulfuric acid hydrolysis of MCC according to Tang’s paper [21]. Briefly, MCC was hydrolyzed in 64% sulfuric at 52 °C for 120 min under ultrasonic treatment at 40 kHz with strong stirring. After hydrolysis, the suspension was washed repeatedly until it become neutralized with distilled deionized water in successive centrifugation (10,000 rpm) and dialyzed against distilled water for 5 days. Finally, the NCC powders were obtained by freeze-drying.

### 2.3. Preparation of KH560-NCC Powders

In a 250-mL three-necked round-bottomed flask equipped with a reflux condenser, a magnetic stirring bar and a dry nitrogen inlet, 0.5 g of NCC powders and 30 mL of dioxane were added. After this, the system was maintained under a dynamic nitrogen atmosphere for 0.5 h in order to create an inert atmosphere. Subsequently, 2.5 g of KH560 and 0.15 mL of triethylamine were added into the flask. Triethylamine worked as a catalyst for the ring-opening reaction [22]. The system was subsequently stirred using a magnetic stirrer at 100 °C for 3 days and maintained under a nitrogen atmosphere. After reaction, the specimen was filtered through 0.22-μm PVDF (polyvinylidene fluoride) filters and washed with dioxane three times. Finally, the KH560-NCC powders were obtained by drying at 60 °C for 2 h.

### 2.4. Preparation of C12-KH560-NCC Powders

A total of 0.5 g of KH560-NCC and DTMOS was added into a round-bottomed flask in a water–ethanol mixture (10 mL of water and 40 mL of ethanol). NaOH and HCl were utilized to adjust the pH of the reaction system. The solution was stirred for the required number of times at room temperature. Finally, DTMOS grafted KH560-NCC (named C12-KH560-NCC) samples were filtered through 0.22-μm PVDF filters, before being washed by deionized water and ethanol for three times, respectively. After this, the obtained C12-KH560-NCC powders were dried at 60 °C for 2 h.

### 2.5. Fourier Transform Infrared (FTIR) Spectroscopy

The FTIR measurement was performed using a Bruker Tensor 27 FTIR spectrophotometer. NCC, KH560-NCC and C12-KH560-NCC samples were analyzed. In detail, 1 mg of samples was mixed with 100 mg of KBr, before being pressed into a pellet and placed in the FTIR spectrometer. The spectra were recorded at a resolution of 4 cm^−1^ in the range of 400–4000 cm^−1^. A total of 32 scans was used to acquire each spectrum.

### 2.6. X-ray Photoelectron Spectroscopy (XPS)

The XPS spectra were recorded on an ESCALAB 250 (Thermo Scientific Instruments Co., Ltd., Waltham, MA, USA) equipped with an Al Kα X-ray source (1486.6 eV). The survey XPS spectra were taken at a pass energy of 100 eV and energy step size of 1 eV, before the carbon surface chemistry was probed with high-resolution regional scans (100 eV pass energy and 0.1 eV step).

### 2.7. HCl–acetone Titration

HCl–acetone titration is a common way to calculate the content of epoxy groups [23]. The HCl–acetone solution was prepared in the volume ratio of HCl to acetone of 1:15. About 0.5 g of the sample was dispersed in 20 mL of HCl-acetone solution. After being stirred for 30 min, 5 drops of the phenolphthalein indicator were added and the excess HCl was titrated by NaOH (0.10 mol·L^−1^). In this paper, this method was applied to determine the presence or absence of epoxy groups. A small difference between the blank consumption and sample consumption of NaOH standard solution will indicate the absence of epoxy groups in the sample.

### 2.8. X-ray Diffraction (XRD)

The crystalline structure and crystallinity index (*C_r_*_I_) changes of the samples were tested by X-ray diffractometer (Rigaku D/Max-Ra, Rigaku, Toyko, Japan) using Cu Kα radiation. The test was conducted in the range of 2θ = 6°–60° at a step size of 0.1°. The *C_r_*_I_ was calculated according to Segal’s method (L Segal 1959). Equation (1) is an empirical method for evaluating the degree of crystallinity in the NCC:

*C_r_*_I_(%) = (*I*_200_ − *I*_am_)/*I*_200_*100 (1)

where *I*_200_ is the maximum intensity of the (200) lattice diffraction peak at 2θ ≈ 22° and *I*_am_ is the lowest intensity at 2θ ≈ 18°, representing the amorphous part of the sample.

### 2.9. Thermogravimetric Analysis (TGA)

The thermal stability of the samples was investigated by TGA (Netasch, TG209 F1, Selb, Germany). The amount of the sample taken for each test was approximately 5 mg. All tests were executed under a nitrogen atmosphere with a gas flow 10 mL/min and heated from 30 to 600 °C at a heating rate of 10 °C/min.

### 2.10. Atomic Force Microscopy (AFM)

The surface morphology of the samples was characterized by Bruker Atomic Force Microscope (AFM) using Nanoscope V, Multimode 8, which was operated in tapping mode and equipped with an integrated silicon tip cantilever with a resonance frequency of 300 kHz.

### 2.11. Water Contact Angle (WCA) Measurement

WCA measurement was executed using the sessile drop configuration at room temperature on the Krüss DSA-30 instrument (Krüss, Hamburg, Germany) equipped with a CDD (Cyclic Delay Diversity) camera. Water droplets of 5 μL were applied in all measurements. The water used was of high purity, which was prepared by a Purescience water purification system. Each sample was tested 3 times, before the average of the data was taken. Both the static contact angle at equilibrium and dynamic contact angle along with time were observed with an angle precision of ±1°.

## 3. Results and Discussion

### 3.1. Fourier Transform Infrared (FTIR) Characterization

**Figure 1:**
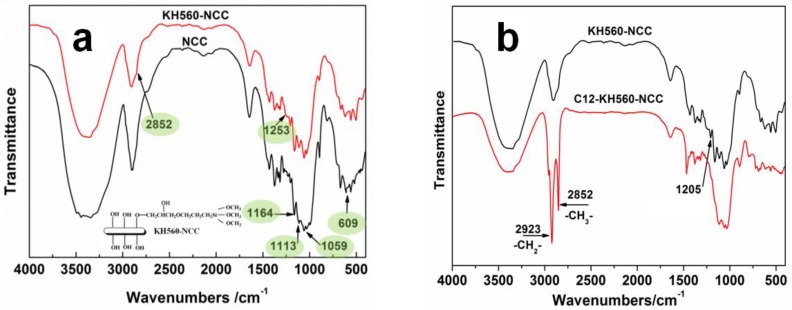
FTIR spectra of NCC and KH560-NCC (**a**), FTIR spectra of KH560-NCC and C12-KH560-NCC (**b**).

The FTIR spectra of the NCC and KH560-NCC are shown in Figure 1a. Both spectra showed absorption bands at 609, 1059 and 1164 cm^−1^, which was attributed to the anomeric carbon, C–O and C–C bands of the NCC backbone [24,25]. Absorption bands at 2988~3707 cm^−1^ were associated with O–H stretching of the NCC structure [26]. We found that for KH560-NCC, the intensity of C–C band at 1164 cm^−1^ is stronger than that of C–O band. In contrast, for NCC, the intensity of C–C band is weaker than that of C–O band. This is because KH560 contains an abundant number of C–C bonds. After introducing additional amounts of KH560, the intensity of absorption bands for C–C increased significantly. The Si–O–C absorption band of KH560 was represented at about 1253 cm^−1^ [27]. Moreover, after KH560 grafting, KH560-NCC had a new small peak at 2852 cm^−1^, which can be attributed to the C–H absorption band of –Si–CH_3_ groups for KH560 [28].

Compared with KH560-NCC, two strong absorption bands at 2923 and 2852 cm^−1^ appeared in the spectrum of C12-KH560-NCC, which were assigned to the stretching vibration of –CH_2_– and –CH_3_ from –C_12_H_25_ chains, respectively. This illustrated that several hydrophobic –C_12_H_25_ chains were successfully introduced to the surface of KH560-NCC, which will be beneficial in the improvement of hydrophobicity of NCC.

### 3.2. XPS Analysis

**Figure 2:**
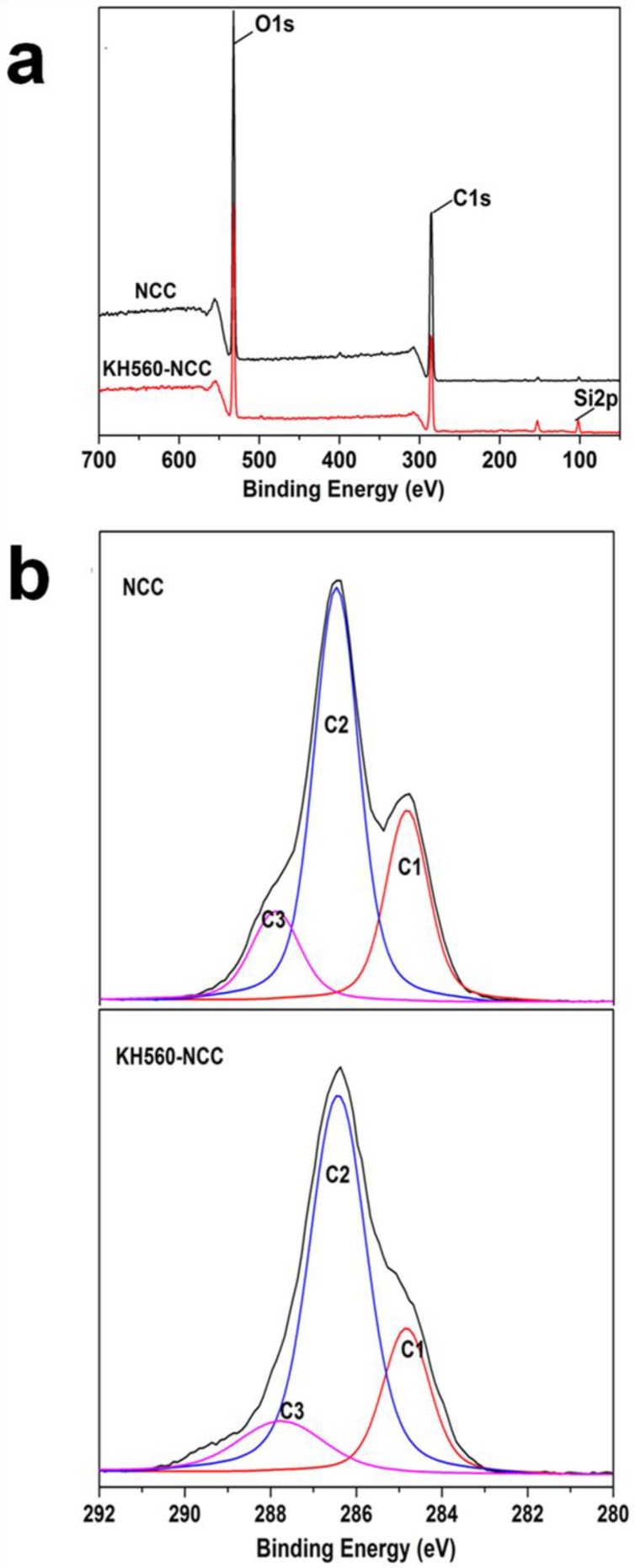
XPS spectra of NCC and KH560-NCC (**a**) and deconvoluted spectra of C1s (**b**).

In order to qualitatively analyze the elemental compositions, XPS was utilized. This spectroscopy is a surface sensitive technique, which provides information in the changes of surface chemistry. Figure 2a indicates the surveyed spectra of NCC and KH560-NCC. It could be seen that both NCC and KH560-NCC samples showed C1s and O1s peaks at 285.17 and 531.17 eV, respectively [29]. For KH560-NCC, there are two new Si2p and Si2s from KH560, which emerged at 101.17 and 152.3 eV. The content of Si increased from 0.00% to 6.25% after KH560 modification. To obtain more information in the changes of the surface compositions, the XPS deconvoluted spectra of NCC and KH560-NCC surfaces were collected in Figure 2b. The high-resolution C1s spectra were resolved into those of various carbon components (Figure 2b). The unmodified NCC show three characteristic functional groups (C1, C2 and C3), in which the C1 peak was assigned to C–C/C–H linkages, C2 peak corresponded to C–O from the alcohols and ethers and finally, C3 peak was attributed to the O–C–O and C=O from the acetal groups [15]. After introducing KH560 into NCC, the intensity of three different carbon components of NCC should have changed. As shown in Table 1, the fraction of C2 increases from 59.7% to 65.61%, while that of C3 decreases from 26.4% to 21.11%. These results indicated that KH560 was successfully introduced to the surface of NCC.

**Figure 3:**
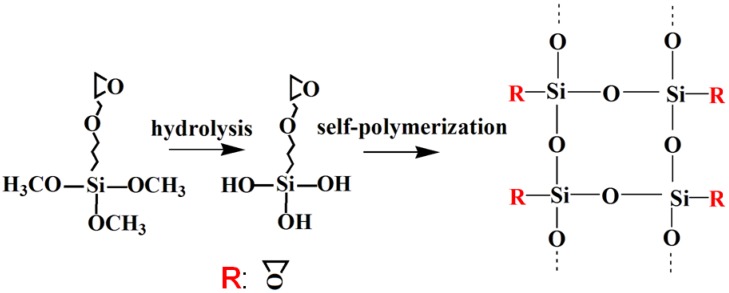
Structural schematic diagram of KH560 with self-polymerization.

The results from FTIR and XPS showed the changes in chemical properties of NCC before and after KH560 modification. However, they could not provide the conclusion that KH560 was covalently grafted onto the NCC surface. This is because the possibility of the absorption of polysiloxane chains, which is a product of self-polycondensation of KH560, onto NCC surface had not been excluded. Figure 3 showed the schematic representation of the self-polycondensation of KH560. After self-polycondensation, polysiloxane possessed several epoxy groups. The epoxy groups can be determined by the HCl-acetone titration method [23]. First, HCl was added into the acetone solution, which contained the test sample. Second, the residual HCl was back-titrated with NaOH solution using phenolphthalein as the indicator.

Table 2 showed the consumption of the NaOH standard solution by the blank and KH560-NCC. The blank consumption of NaOH standard solution is both 7.2 mL for the acetone and HCl solution with and without unmodified NCC, while the sample consumption of NaOH standard solution is 7.5 mL. This indicates convincingly that there is no epoxy group in KH560-NCC. Compared with the blank, the additional consumption of NaOH standard solution is probably due to the hydrolysis of –Si–OCH_3_ during titration. Therefore, combined with results from FTIR, XPS and HCl–acetone titration, it can be concluded that KH560 was covalently grafted onto the surface of NCC by C–O–C bonds after a ring-opening reaction. Finally, according to the reported method, the substitution rate of KH560 onto NCC was calculated to be 2.4 [9,30].

### 3.3. XRD Analysis

**Figure 4:**
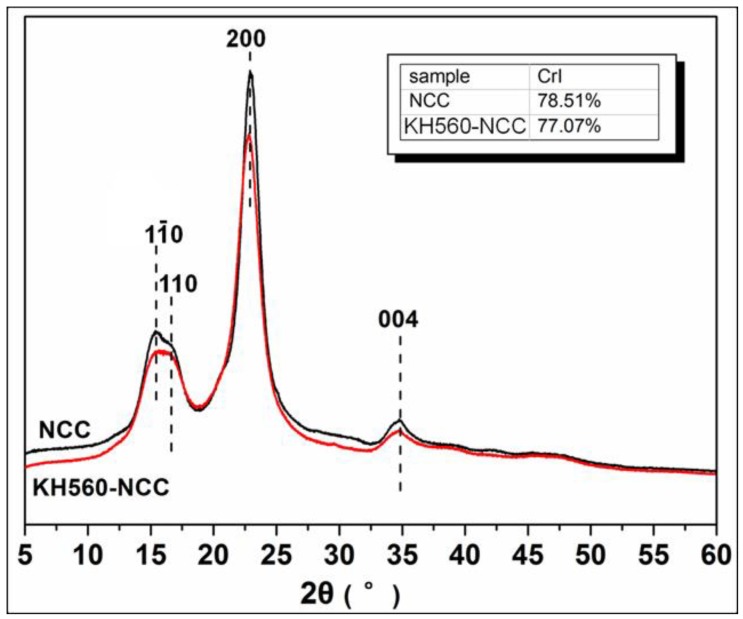
XRD patterns of NCC and KH560-NCC.

The crystalline structure of the KH560-NCC and NCC samples were analyzed by XRD (Figure 4). All samples had similar characteristic diffraction peaks at 2θ values of around 15.2°, 16.2°, 22.8° and 34.7°, which corresponds to the 1$\bar{1}$0, 110, 200 and 004 diffraction planes of cellulose type I [8,15]. After being grafted with KH560, the crystalline structure of NCC samples did not change obviously. The crystallinity of NCC before and after KH560 modification is tabulated in the inserted map of Figure 4. It could be seen that the KH560 modification resulted in a very slight decrease in the NCC crystallinity.

### 3.4. AFM Analysis

**Figure 5:**
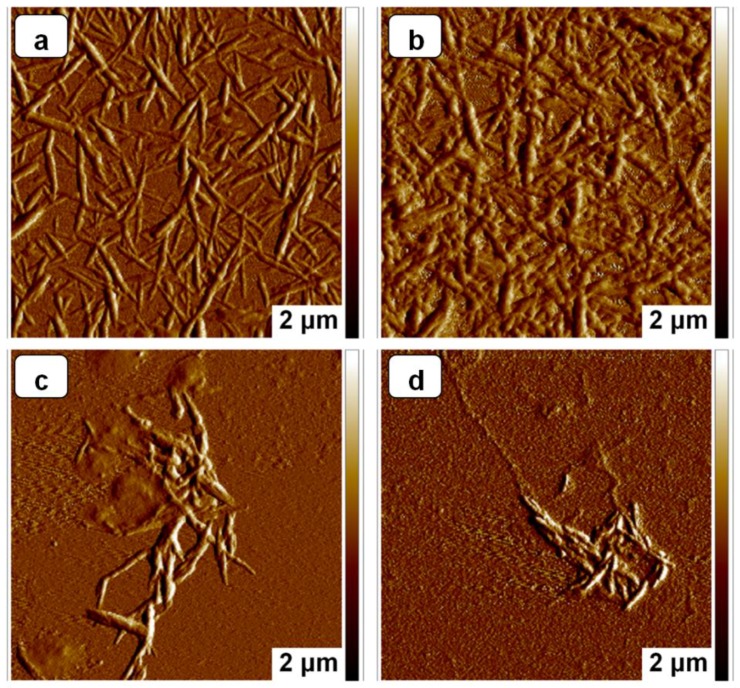
AFM images of NCC before (**a**) and after (**b**) freeze-drying, KH560-NCC (**c**) and C12-KH560-NCC (**d**).

Atomic force micrographs (AFM) were used to observe the morphology and measure the dimensions of NCC. AFM images of the samples from NCC hydrogel, freeze-dried NCC powders, KH560-NCC and C12-KH560-NCC are shown in Figure 5. The individualized rod-like particles and a classical web-like network structure could be seen in the Figure 5a. It could be seen that the surface of the NCC hydrogel was smooth, while the freeze-dried NCC powders were rougher (Figure 5a,b). Furthermore, the geometric average length and diameter of NCC hydrogel were around a few hundred nanometers and 58.22 nm, respectively, while the diameter of freeze-dried NCC powders was increased to around 62.75 nm. These results were attributed to the effect of the interactions through hydrogen bonding, which leads to severe aggregation. After KH560 modification and further DTMOS modification, the diameter of NCC was further increased to around 67.40 and 76.20 nm, respectively. From the dimensional changes, it was shown that the average diameter gradually increased for KH560-NCC and C12-KH560-NCC due to the introduction of grafting molecular chains onto the surface of NCC.

### 3.5. Hydrophobicity

To realize modified NCCs with good hydrophobicity, the effect of pH value, weight ratio of modifier to NCC and reaction time on the water contact angles (WCA) was investigated.

**Figure 6:**
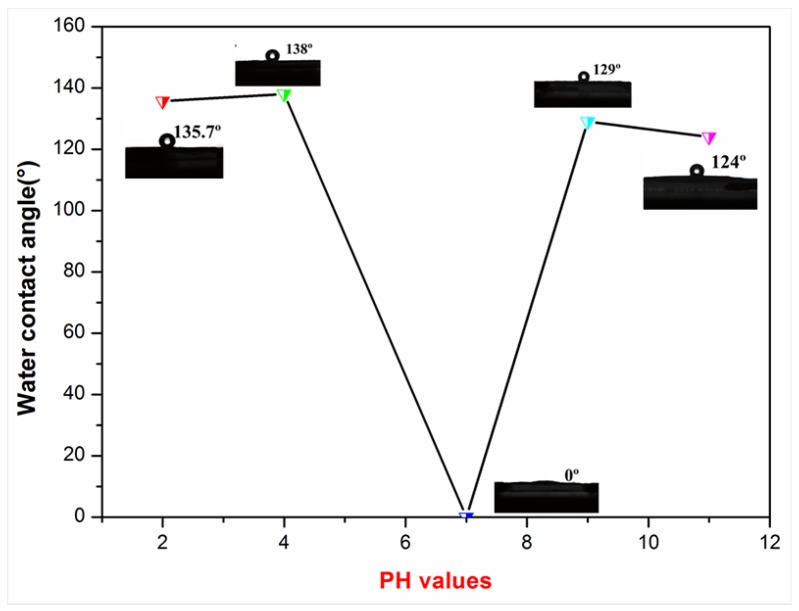
Effect of pH on the WCA of C12-KH560-NCC powders.

Figure 6 showed the change in the WCA of C12-KH560-NCC as a function of pH value. Almost all samples were found to have excellent hydrophobicity except for the sample produced with a pH under 7. This result could be well-understood because that silane did not hydrolyze or slowly hydrolyze under neutral conditions. It was also noteworthy that the hydrophobicity of samples with acidic catalysts (pH2 and pH4) was better than basic catalysts (pH9 and pH11). Therefore, the pH value was optimized to be 4.

**Figure 7:**
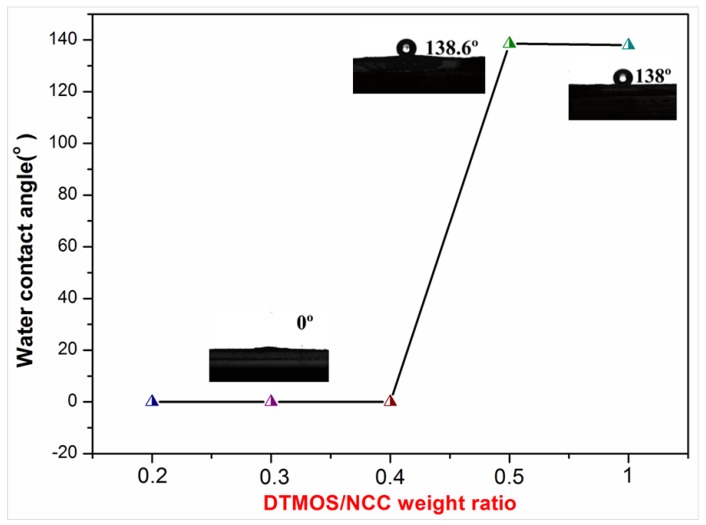
Effect of modifier content ratio on the WCA of C12-KH560-NCC powders.

Figure 7 showed the effect of the weight ratio of DTMOS to NCC on the WCA of C12-KH560-NCC. As shown in Figure 7, as the weight ratio increased from 0.2 to 0.4, the WCA was almost unchanged with increasing DTMOS content. A further increase in modifier content resulted in an obvious increase in WCA. When the modifier content ratio was 0.5, the WCA of C12-KH560-NCC was increased to 138.6°. The weight ratio of modifier to NCC was selected to be 0.5.

**Figure 8:**
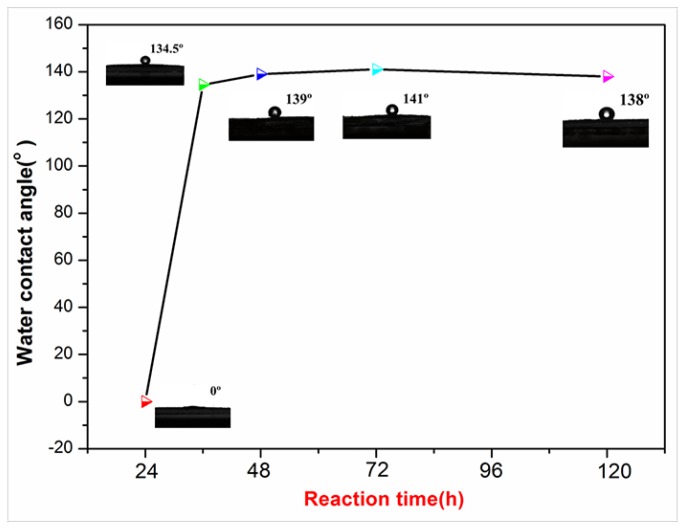
Effect of reaction time on the WCA of C12-KH560-NCC powders.

Figure 8 showed the change in the WCA of C12-KH560-NCC as a function of reaction time. It could be seen that the hydrophobicity of C12-KH560-NCC improved drastically as the reaction time increased from 24 to 36 h. The WCA of C12-KH560-NCC increased significantly from 0° to 134.5°. This is probably because the hydrolysis reaction of alkoxy groups was slow. As the reaction time increased from 36 to 48 h, the WCA increased to 139°. A further increase in reaction time did not result in an obvious increase in WCA. Thus, the reaction time was optimized to be 48 h.

### 3.6. Thermal Analysis

**Figure 9:**
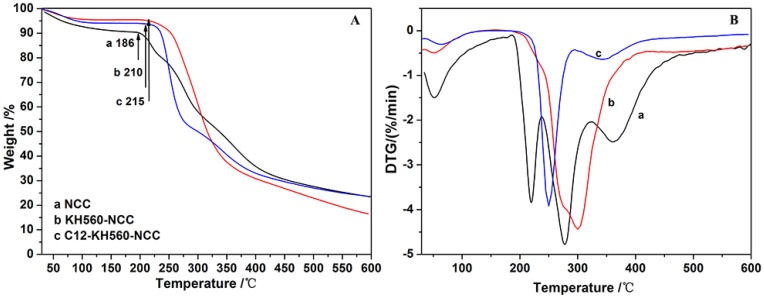
TG curves (**A**) and DTG curves (**B**) of NCC (**a**), KH560-NCC (**b**) and C12-KH560-NCC (**c**).

Thermal stability is an important physical property of NCC, which plays a vital role in the fabrication of melt processed NCC-reinforced composites [8]. Pure NCC has poor thermal stability and therefore, thermal degradation might occur during the processing of composites at a high temperature, particularly under the circumstances of thermal extrusion and hot compression processes. The silane modification of NCC could affect the thermal stability of NCC. Therefore, it was necessary to study the thermal stability of NCC before and after KH560 modification and a further DTMOS modification. Figure 9 showed the TGA and DTG curves of NCC, KH560-NCC and C12-KH560-NCC. As shown in Figure 9a, NCC showed a weight loss from room temperature to temperatures of 150 °C. It was ascribed to the evaporation of absorbed water. This effect was decreased for the KH560-NCC and C12-KH560-NCC due to a lower accessibility of –OH groups after the grafting reaction, as shown in Figure 9b,c. In addition, NCC showed the decomposition behavior with the onset degradation temperature (*T*_onset_) of about 186 °C. Compared with NCC, KH560-NCC and C12-KH560-NCC exhibited a relatively higher *T*_onset_ as they began to degrade at 210 and 215 °C, respectively (Table 3). These results indicate that the KH560-NCC and C12-KH560-NCC both had an enhanced thermal stability.

## 4. Conclusions

In summary, we developed a two-step silanation method for surface hydrophobic modification of NCC with enhanced thermal stability. FTIR, XPS and HCl-acetone titration analysis results revealed that the KH560 and DTMOS were successfully grafted to the surface of NCC by covalent bonding. The WCA of the NCC samples increased remarkably after KH560 and a further DTMOS modification, which indicated that the proposed treatment effectively improved the hydrophobicity of NCC. The starting temperature of the thermal degradation of KH560-NCC and C12-KH560-NCC samples was higher than pure NCC, which suggested that the two-step modification method could enhance the thermal stability of NCC.

## Figures and Tables

**Table 1:** XPS analysis of NCC and KH560-NCC.

Sample	Elemental Analyses (%)			Binding Energy (eV)		
	C	O	Si	C1, 285 ± 0.1 C–C/C–H (%)	C2, 286.4 ± 0.1 C–O (%)	C3, 287.7 ± 0.1 O–C–C/C=O (%)
NCC	59.87	40.12	0	26.4	59.7	13.9
KH560-NCC	55.58	38.18	6.25	21.11	65.61	13.28

**Table 2:** The consumption of NaOH standard solution of blank and KH560-NCC.

Sample	Sample Detail	V_NaOH_ (mL)
blank 1	acetone and HCl	7.2
blank 2	acetone, HCl and 0.5053 g of NCC	7.2
sample 1	acetone, HCl and 0.5024 g of KH560-NCC	7.5

**Table 3:** Degradation data obtained from TGA for NCC, KH560-NCC and C12-KH560-NCC: *T*_onset_ and *T*_max_ corresponds to the beginning of the degradation process and the thermal decomposition temperature associated with the maximum derived signal, respectively.

Sample	*T*_onset_/°C	*T*_max_/°C
NCC	186	278
KH560-NCC	210	300
C12-KH560-NCC	215	250
